# Behavioural Type Affects Space Use in a Wild Population of Crows (*Corvus corone*)

**DOI:** 10.1111/eth.12536

**Published:** 2016-10-06

**Authors:** Sarah A. Deventer, Florian Uhl, Thomas Bugnyar, Rachael Miller, W. Tecumseh Fitch, Martina Schiestl, Max Ringler, Christine Schwab

**Affiliations:** ^1^ Department of Cognitive BiologyUniversity of ViennaViennaAustria; ^2^ Department of PsychologyUniversity of CambridgeCambridgeUK; ^3^ Department of Linguistic and Cultural EvolutionMax Planck Institute for the Science of Human HistoryJenaGermany; ^4^ Department of Integrative ZoologyUniversity of ViennaViennaAustria; ^5^ Messerli Research InstituteUniversity of Veterinary Medicine ViennaMedical University of ViennaUniversity of ViennaViennaAustria

**Keywords:** personality, coping style, space use, docility, tonic immobility, antipredator

## Abstract

While personality‐dependent dispersal is well studied, local space use has received surprisingly little attention in this context, despite the multiple consequences on survival and fitness. Regarding the coping style of individuals, recent studies on personality‐dependent space use within a habitat indicate that ‘proactive’ individuals are wider ranging than ‘reactive’ ones. However, such studies are still scarce and cover limited taxonomic diversity, and thus, more research is needed to explore whether this pattern generalises across species. We examined the link between coping style and space use in a population of crows (*Corvus corone*) freely inhabiting the urban zoo of Vienna, Austria. We used a binary docility rating (struggle during handling vs. no struggle) and a tonic immobility test to quantify individual coping style. Individual space use was quantified as the number of different sites at which each crow was observed, and we controlled for different number of sightings per individual by creating a space use index. Only the binary docility rating showed repeatability over time, and significantly predicted space use. In contrast to previous studies, we found that reactive crows (no struggle during handling) showed wider ranging space use within the study site than proactive individuals (who struggled during handling). The discrepancy from previous results suggests that the relationship between behavioural type and space use may vary between species, potentially reflecting differences in socioecology.

## Introduction

Selection acts on variability in a population, and thus, understanding consistent differences in the behaviour of individuals, often referred to as ‘behavioural syndromes’, ‘temperament’ or ‘personality’, substantially contributes to our knowledge of species ecology (Réale et al. 2007). One of the dimensions along which animals have been suggested to differ is their ‘coping style’ (Koolhaas et al. 1999, 2010), also termed ‘responsiveness’ (Wolf et al. 2008). These terms refer to how individuals cope with stressors, on a behavioural continuum between two opposing coping strategies: being proactive or reactive. Behaviourally, proactive individuals are characterised by taking action to end or escape a stressful situation, while reactive individuals are characterised by freezing behaviour and immobility when confronted with a stressor (Koolhaas et al. 1999). Further, proactive individuals appear to be rather unresponsive to external stimuli and quickly form routines that are rigidly followed without perturbation by changed environmental cues. Reactive individuals respond more flexibly to short‐term changes in the environment (reviewed in Koolhaas et al. 1999 and Wolf et al. 2008). These differential coping styles are often associated with a set of further behavioural characteristics: proactive individuals tend to be more bold, active, aggressive and explore faster although also more superficially than reactive individuals (Koolhaas et al. 1999; Carere et al. 2010).

Given the ubiquity of consistent individual differences in behaviour (Bell et al. 2009), the evolutionary ecology of this variation in behavioural type has been subject to much theoretical and experimental work, especially over the last decade (overview in Réale et al. 2010 and Wolf & Weissing 2012). In the context of fitness consequences of different behavioural types, studying personality‐dependent spatial movement within or between habitats is fundamental, because it is ecologically linked to multiple fitness‐relevant factors. The choice of habitat and spatial use of a habitat determine an individual's access to resources, such as food (Stephens & Krebs 1986) and social partners (Brown & Orians 1970). It further influences predation risk (Lima & Dill 1990), as well as the acquisition and spread of both information and pathogens (Barber & Dingemanse 2010; Boyer et al. 2010). If coping styles influence the way animals use and perceive their environment, we expect a direct effect of these differences on their habitat use, with fitness‐relevant consequences.

To date, most studies concerning personality‐dependent habitat use have focused on the link between behavioural type and dispersal, generally finding that proactive individuals, measured in boldness and aggression, show higher dispersal tendencies (e.g. Trinidad killifish: Fraser et al. 2001; great tits: Dingemanse et al. 2003; western bluebirds: Duckworth & Badyaev 2007; reviewed by Cote et al. 2010). Only recently, a few studies have started to investigate whether behavioural type affects local space use, the issue that we address in this study. Great tit families with females that were more superficial explorers had larger home ranges (van Overveld et al. 2011). In the same great tit study population, superficial explorers travelled further distances after experimental removal of feeders than slow and thorough explorers (van Overveld & Matthysen 2010). Likewise, individual juvenile starlings’ home range size was predicted by their exploration behaviour in a novel environment (Minderman et al. 2010). In North American red squirrels, females scoring high in an activity essay were trapped in a greater number of locations than females that exhibited low activity (Boon et al. 2008). Siberian chipmunks showed the same link: individuals that scored high on an exploration‐activity axis were trapped in a larger number of different sites (Boyer et al. 2010). Thus, the picture is emerging that individuals with a proactive coping style show wider ranging space use than reactive ones. However, studies on this connection are still scarce, and of limited taxonomic diversity. More research is thus needed to explore whether we can generalise this finding across species with varying social systems and feeding ecology.

Here, we studied a wild population of crows to test whether and how coping style affects individual local space use. The study population comprises both carrion (*Corvus corone corone* L.) and hooded (*Corvus corone cornix* L.) crows, and hybrids of these two subspecies that have overlapping ranges in central Europe (Melde 1984). We refer to both subspecies and hybrids as ‘crows’ hereafter. Although crow breeding pairs are somewhat sedentary once they occupy a territory (Charles 1972; Melde 1984), the majority of crows of all age classes are mobile non‐breeders. These exhibit considerable variation in movement patterns and a flexible social system characterised by fission–fusion dynamics (Melde 1984; von Blotzheim & Bauer 1993; Charles 1972; Sonerud et al. 2002). The research was conducted in the urban zoo of Vienna (Zoo Vienna at Schönbrunn), which provides exceptional foraging opportunities, drawing in large numbers of crows well‐habituated to humans, and thus rendering it an excellent site for our project. Crows are omnivores, and apart from using the natural food sources in the zoo, feed on food provided for zoo animals, as well as on leftover food from zoo visitors (Miller et al. 2014). The frequency of visits to the zoo varies considerably between individual crows, ranging from crows that are only sighted once, to crows encountered regularly. Crows use the zoo to forage, socialise and breed and can be observed within, as well as outside of animal enclosures. The zoo thus provides a well‐defined habitat where the space use of crows during foraging is easily observed.

We experimentally assessed the coping style of wild crows during a brief handling process after being trapped for ring marking, before release. We subsequently recorded and evaluated their space use within the zoo based on the number of sites where an individual was encountered (cf. Boon et al. 2008; Boyer et al. 2010). This follows the rationale that individuals that use more sites have a wider ranging space use. We conducted two studies in separate years, following different sampling regimes. In study 1, we quantified the space use of individual crows based on the number of pre‐existing foraging sites they utilised within the zoo. Study 2 aimed at replicating study 1 with a higher sampling effort, and to quantify crows’ space use with an evenly spaced grid of encounter sites.

Although our main focus in this study was the relationship between behavioural differences and habitat use of individuals, we also controlled for individual attributes of crows that could affect their habitat use: dominance, (indicated in crows by sex and age; Richner 1989), body condition, phenotypic degree of hybridisation and breeding/non‐breeding status.

## Methods

### Study Site

The project was conducted in the urban zoo of Vienna, Austria (‘Tiergarten Schönbrunn’, 48°10′56″N, 16°18′10″E). We surveyed crows throughout the zoo in an area of 11.887 ha, but spared a forested area that differed from the rest of the zoo in three respects: a lack of animal enclosures and therefore foraging sites, reduced visibility of crows and limited observer access on the few trails in this part of the zoo (Figure S1).

### Capture and Handling Procedure

Crows for ringing and testing were caught from Feb. to Aug. 2012 using baited ladder drop‐in traps (Kalmbach 1939) placed on the zoo premises (permit number MA 22‐234375/2013; issued by the Municipal Department for Environmental Protection (MA 22) Vienna, Austria). During these periods, traps were active one to 4 d per week and checked every hour. Trapped birds were removed from the trap sequentially and handled immediately by one of three different handlers (SD, RM and CS). The handling procedure lasted around 15 min per crow. We caught a total of 130 crows and obtained coping style data for 112 of them (in 18 cases timing did not allow for completion of tests). These 112 crows were considered for inclusion in this study, but subjected to additional selection criteria as described further below, yielding a total sample size of 36 crows.

During handling, we fitted birds with unique leg rings. For DNA sexing, we collected at most 600 μl blood from the wing vein (permit number for blood sampling: BMWF‐66.006/0009‐II/3b/2012, issued by the Federal Ministry of Science and Research, Austria). Crows reach maturity around the age of 3 yr. By assessing colouration of the oral cavity and plumage, three age classes can be distinguished in crows with unknown history: hatched in the previous year, hatched 2 yr ago and hatched three or more years ago (adult) (Svensson 1992; von Blotzheim & Bauer 1993). Accordingly, we classified crows into three categories: hatched in 2011, 2010 and before 2010.

Further, we established the body condition of birds, as poor condition may trigger increased foraging behaviour (Belthoff & Dufty 1998), which could impact space use. We weighed birds and measured tarsus length as a linear indicator of size that is stable over time irrespective of nutritional status and remains constant from fledging (Richner 1989). A body condition index was then calculated as the residual from the regression between weight and the cube of tarsus length, using separate regressions for each sex to account for sexual size dimorphism (Potti 1993; Marcos & Baglione 2003).

Previous studies on the two crow subspecies in other areas of the European hybrid zone indicate differences in habitat preferences between the subspecies and the hybrid forms (Saino 1992; Randler 2007). We therefore visually estimated the degree of hybridisation of each crow by phenotype (from here on referred to as ‘hybridisation index’), on a scale from one (pure carrion crow) to five (pure hooded crow), following the categorisation of Rolando (1993).

### Coping Style Assessment

The coping style of individuals was assessed during and after the ringing procedure. The coping style of an animal reflects its short‐term reaction to a stressor, such as confrontation with a predator. We used two established tests to assess crows’ behavioural coping style: a docility rating and a tonic immobility test (e.g. Erhard et al. 1999; van den Brink et al. 2012; Edelaar et al. 2012). Docility refers to how much an individual struggles when handled by a human and is thought to reflect antipredator behaviour (Réale et al. 2000). Docility is often quantified on a Likert‐like scale (Likert 1932) through a rating of the amount of struggle an animal shows during handling (Brommer & Kluen 2012: ‘handling aggression’ in their terminology; van den Brink et al. 2012). As most birds in our population did not struggle during handling, we adopted a simplified binary docility rating, distinguishing only immobility (0 = considered reactive, n = 28) and struggle (1 = considered proactive, n = 8), that is frequent movements, pecking or biting.

After the handling procedure and docility rating, crows underwent a tonic immobility test. Tonic immobility (TI) is a behavioural state of immobility in which animals appear to feign death (Gallup 1974). It is an antipredator behaviour that can be induced in a wide range of vertebrates and invertebrates by a short physical restraint, usually in a dorsal or lateral position (Sargeant & Eberhardt 1975). However, individuals of the same species may vary in whether or not the TI state can be induced. In addition, individuals with induced TI may vary in the latency to right themselves back to a regular body posture (i.e. duration of the TI) (Gallup 1974). Handlers positioned each crow flat on its back and subsequently released their grip. The majority of birds then instantly righted themselves, indicating no TI. Other individuals showed TI by remaining immobile on their back (Video S1). Of the birds showing TI, only two righted themselves within 20 s, while we righted the other birds manually after a cut‐off time of 1 min as a precaution against detrimental effects such as temperature loss. We thus binarily classified the successful induction of TI (0 = considered reactive, n = 12) or the lack thereof (1 = considered proactive, n = 24). After righting, birds were not visibly affected by the immobile period and flew off.

### Repeatability of Coping Style Measures

Because of low recapture rates in 2012, we used additional coping style data from a previous study that was performed in 2010 (M. Schiestl, unpublished data), as well as re‐catches from subsequent years (2013/2014) to calculate repeatability of coping style measures over time. Coping style tests in each year were conducted in a directly comparable manner which was coordinated by RM who took part in both studies. A subset of 21 crows entered the traps twice; either twice in 2012 or once in 2010 and once in subsequent years (2012–2014). Because many crows could only be tested once, we only used results from the first test round (in case they were tested twice in 2012) or the most recent test (in case they were tested previously in 2010) in further analyses.

### Breeding Status

We assessed the breeding/non‐breeding status of individuals in the years with concurrent space use observations. We observed nesting activity during the breeding season (Mar. to May) in 2013 and 2014. All nests on zoo premises were mapped and their development monitored. Birds were considered breeders when they were observed to be incubating (females) or feeding the incubating female (males).

### Overview of Space Use Assessment

Our logic followed the method used by Boon et al. (2008) and Boyer et al. (2010), in which the number of trapping locations of an individual was used as an indicator of its space use, with a higher number of trapping locations implying a wider ranging space use. However, because crows are hard to catch but quite easy to observe, we used sightings instead of trappings. To assess their space use, we thus counted the number of different sites that individuals were sighted at and calculated a space use index. We used two different methods to define discrete sites in our study area. Additionally, we calculated kernel density estimates (KDE) (Worton 1989) based on individual sighting locations of crows.

#### Foraging site method

In the first method, we used 38 pre‐existing sites in the zoo that were frequented by the crows for foraging and offered good feeding opportunities (Figure S1). Of these, 33 sites were animal enclosures in which crows could access fodder provided for zoo animals, such as grains, vegetables and meat. Further, there were five non‐enclosure foraging sites accessible to zoo visitors with outdoor restaurants and food stalls, where crows fed on visitor leftovers. Sites thus differed from each other in the type of food available, vegetation and layout as consequences of accommodating captive zoo animal species or human visitors. The zoo species also varied in their behaviour and potential threat to the crows. Moreover, topographic features such as fences, paths and houses had previously been observed to influence the habitat use of crows (Wittenberg 1968). We therefore considered these 38 sites a biologically relevant division for the crows.

#### Grid site method

Further, to apply a more objective and regular method, we overlaid a regular grid (cf. Boon et al. 2008; Boyer et al. 2010) of hexagonal sites onto the zoo area using qgis 2.6 (QGIS Development Team 2016) (Figure S2)**.** To allow direct comparisons with the ‘foraging site method’, we scaled the hexagons (0.3115 ha) to obtain the same number (38) of grid sites as the foraging sites across the zoo. We only used hexagons that overlapped with the study area by at least 50 per cent, resulting in truncation of some peripheral hexagons (n = 17/38, size range = 0.1902–0.3109 ha).

#### Space use index

To control for variation in the number of sightings between birds, we implemented a space use index that applied for both the ‘foraging site method’ and ‘grid site method’. This index was calculated by dividing the ‘observed number of sites’ used per bird by *‘*the number of sites we would expect’ each bird to be seen in if it were visiting sites at random, given its number of sightings. To calculate the ‘expected number of sites’ (of 38) under random choice, we used probability combinatorics with item replacement based on the number of sightings per individual crow. The space use index was thus calculated as follows:

$$\begin{array}{cc} \text{space use index} & =\frac{\text{observed no. of sites}}{\text{expected no. of sites}}\\ & =\frac{o}{n\frac{n^{k}-{\left(n-1\right)}^{k}}{n^{k}}} \end{array}$$

with *o* = observed number of different sites visited by a crow, *n* = total number of distinct sites (=38 sites) and *k* = the number of sightings of that particular crow. The closer the space use index is to one, the closer the match between observed and expected number of sites. If a crow was seen in more sites than expected by chance, the number is above one. If it was seen in fewer sites than expected, the number is below one.

#### Kernel density estimation

The kernel density estimation (KDE) (Worton 1989) is a common method to estimate individual home ranges and has previously been used in studies on personality‐dependent space use in other bird species (Minderman et al. 2010; van Overveld et al. 2011). KDE is best suited for large numbers of sightings per individual such as obtained by radio telemetry. Our space use index is more robust for smaller sample sizes as it controls for the number of sightings. However, because of its wide use in the literature, KDE provides a more comparative measure and additionally verifies our space use index.

We used fixed kernel density estimation as implemented in the Home Range Tools (HRT) (Rodgers et al. 2007) extension for ArcGIS^®^ 9.3 (ESRI). Smoothing parameters were estimated with the hRef method. For subsequent analyses, we used the 50% contour of the utilisation distribution. Note that while KDE is commonly used to calculate entire home ranges, here we used this measure to estimate the utility distribution of individual crows only within the zoo, where our sampling took place.

### Space Use Data Collection

#### Study 1

In study 1, we used the foraging site method to relate individual space use to coping style. Observations in the zoo took place over 7 mo, between Mar. and Oct. 2013. We counted in how many different foraging sites, each individual was sighted. During sampling (n = 23 d), the observer (SD) haphazardly visited all 38 sites repeatedly and identified marked birds. Revisits to particular sites were at least 1 h apart. We also included incidental sightings of marked crows (by SD, RM and CS) that occurred while conducting other non‐invasive research in the zoo during the study period (contributing 17% of overall sightings).

We only included birds with at least eight sightings in the analysis, as a compromise between sample size and accuracy of space use estimation. Our final sample size for study 1 consisted of 25 crows.

#### Study 2

In the second study, we aimed at replicating study 1 with more observations and a more standardised sampling scheme. In addition to using the foraging site method, we quantified crows’ space use using the regular grid site method and calculated KDEs for verification. As individuals with different behavioural types may differ in their propensity to be influenced by social context in their space use (Aplin et al. 2014), we counted the number of other crows (marked or unmarked) present within a 5‐m radius of the focal individual for each sighting.

Instead of visiting specific sites, observations (by FU) were performed continuously along a defined line transect covering the entire study area in the zoo to standardise sampling effort. Starting and end points were alternated. Locations of sighted crows were entered into a detailed map of the zoo using a handheld GPS enabled mapping device (MobileMapper^®^ 10, Spectra Precision) with a mobile GIS software (ArcPAD^®^ 10.2, ESRI). Surveys were conducted on 122 d (two to three times per week) between Jan. 2014 and Jan. 2015. Sampling was performed 2× per day in the winter months (morning and afternoon) and 3× per day during summer months (morning, afternoon and evening). Slight variation of the transect occurred when access to certain sections was temporarily prohibited during winter.

The mapped crow locations were then overlaid with the ‘foraging sites’ and ‘grid sites’, respectively, using qgis, to obtain the number of different sites each crow was observed in (Figure S3). KDEs for each crow were calculated in ArcGIS.

Further, we used these data to address a potential difference in the extent to which individuals with different coping styles may use the zoo. If individuals of one behavioural type are more likely to have home ranges that lie predominantly outside of the zoo area, we may underestimate their space use. Such a bias should be apparent by these individuals being sighted rather at the periphery of the zoo. Therefore, we calculated the average distance of an individuals’ sightings to the closest point in the zoo boundary, as well as the proportion of each crow's KDE that lay outside of the zoo boundary as an indicator of the KDE's overlap with the zoo boundary. We then tested whether these measures differed between proactive and reactive crows.

Using the same sightings threshold of a minimum of eight sightings per bird, the final sample size for study 2 consisted of 28 crows.

As crows tended to visit the zoo over multiple years, 17 crows had a sufficient number of sightings in both studies. Thus, a total of 36 individuals provided the data for this study, eight of which were unique to study 1 and 11 unique to study 2. However, while the coping style of individuals was assessed only once, space use was evaluated independently in each study, and in separate years. No data regarding the space use of individuals were shared between the two studies. Furthermore, the observer of study 2 (FU) did not take part in study 1 (observers: SD, RM and CS) and was blind to individual crows’ previous data.

### Statistical Analyses

For birds with two behavioural‐type test rounds, repeatability estimates of TI and docility scores were calculated following the guidelines of Nakagawa & Schielzeth (2010) for binary data. We fitted mixed models using the package ‘MCMCglmm’ (Hadfield 2010) (5 300 000 iterations with 300 000 burn‐in rate, thinning interval = 1000) with subject identity as a random effect and test sequence (first or second) as a fixed effect. In these models, we used uninformative priors (inverse Gamma).

We considered the influence of multiple factors on the space use of crows by pooling the data of both studies and building a generalised linear mixed model (GLMM) with space use index as the dependent variable, using the r package ‘lme4’ (Bates et al. 2015). We square root‐transformed the space use index to improve normality. As fixed effects, we entered docility, age, sex, body condition, hybridisation index, breeding status and type of site (foraging site or grid site) used to calculate the space use index. As random effects, we entered subject ID and study (study 1 or study 2). As the accuracy of an animal's space use estimation improves with an increasing number of relocations, we weighted cases by the number of sightings per individual. To examine the influence of each fixed effect, we used a likelihood ratio test by comparing the models with and without the fixed effect of interest with each other. To avoid cryptic multiple hypothesis testing, we refrained from model selection procedures (Forstmeier & Schielzeth 2011). We considered birds to be breeders when they were observed breeding in the year of data acquisition (2013 for study 1 and 2014 for study 2).

To test whether the number of crows surrounding the focal crow (≤5 m radius) at the time of sighting differed between proactive and reactive crows, we fitted a negative binomial GLMM using the r package ‘lme4’ (Bates et al. 2015), with the number of surrounding crows as the dependent variable, docility as fixed effect and individual as random effect. Additionally, time of day (morning, noon or afternoon) and date were added as random factors, as these factors may influence the number of crows present in the zoo. Using the same r package, we also fitted a binomial GLMM with a binary dependent variable indicating whether crows were seen alone or with at least one other crow (within ≤5 m radius). Again, we entered docility as a fixed effect, and individual, time of day and date as random effects.

Repeatability and GLM modelling was conducted in R 3.3.1 (R Core Team 2016); all other statistical tests were performed in ibm spss ^®^ (version 19.0.0). All tests were two‐tailed, and α was set to <0.05.

## Results

Repeatability of responses were *r* [95% CI] = 0.968 [0.201–0.999] n = 20 for docility, and 0.002 [<0.001–0.886] n = 21 for TI. This suggests that the docility as measured here is indeed a stable property of crows in our population. In contrast, response to the tonic immobility test was not repeatable. Consequently, we only present the results of analyses involving the docility scores. The frequency distribution of docility and TI scores for all birds considered in this study is given in supplementary material (Table S1).

In study 1, a total of 336 sightings were recorded for 25 birds with a $\overset{¯}{x}$ ± SE of 13.44 ± 1.04 sightings per bird (range: 8–24). In study 2, a total of 1004 sightings were recorded for 28 birds, with a $\overset{¯}{x}$ ± SE of 35.86 ± 5.57 sightings per bird (range: 8–128). In study 1, the number of sightings did not differ significantly between the behavioural types (Mann–Whitney *U*‐test, docility: *U* = 56.5, p = 0.511, n = 25). In study 2, proactive crows (docility score 1) were sighted considerably more often ($\overset{¯}{x}$ ± SE = 73.75 ± 23.83) than reactive ones (docility score 0) ($\overset{¯}{x}$ ± SE = 29.54 ± 4.24; Mann–Whitney *U*‐test: *U *=* *17.0, p = 0.041, n = 28).

### Space Use Index

**Figure 1:**
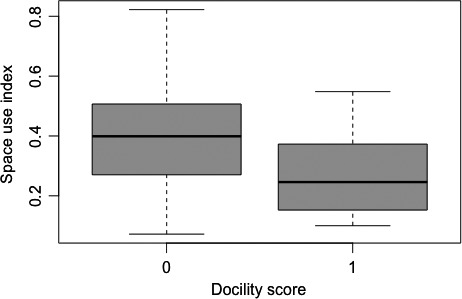
Crows with a docility score of 0, representing an immobile, reactive response during handling (n = 28), have higher space use values than crows with a docility score of 1, representing a struggling, proactive response during handling (n = 8).

Docility was the only variable that significantly affected space use (χ^2^(1) = 6.91, p = 0.009), increasing the space use index by about 0.13 ± 0.04 for reactive individuals compared to proactive ones (Fig. 1). Interestingly, the space use index was below one for all birds, suggesting that all birds used fewer sites than expected by chance (Table 1).

**Table 1:** Results of study 1 and study 2 for the space use behaviour of crows based on the foraging and grid site methods. Note that the grid site method was not available for study 1

	Foraging site method				Grid site method			
	Sites visited		Space use index		Sites visited		Space use index	
	Mean ± SE	Range	Mean ± SE	Range	Mean ± SE	Range	Mean ± SE	Range
Study 1	4.68 ± 0.36	2–8	0.43 ± 0.03	0.19–0.74				
Study 2	5.32 ± 0.41	2–10	0.30 ± 0.03	0.07–0.61	7.0 ± 0.5	3–14	0.39 ± 0.03	0.14–0.82

### Kernel Density Estimates

Consistent with results from the space use indices, kernel density estimates (utilisation distributions) were significantly larger for reactive individuals with a docility score of 0 compared to proactive individuals with a docility score of 1 (Mann–Whitney *U*‐test, 50% KDE: *U *=* *17.0, p = 0.042, n = 28).

### Location of Sightings

The average distance of sightings from the closest point in the zoo boundary did not differ between individuals with different coping styles (*t*‐test: *t* = −0.004, p = 0.997). There was also no difference between the behavioural types in how peripherally their KDEs were located, as indicated by the proportion of individuals’ KDEs that lay outside of the zoo area (Mann–Whitney *U*‐test: *U* = 42.0, p = 0.655).

### Social Context

The number of crows in the immediate vicinity (≤5 m) of focal crows did not differ between individuals with different coping styles (negative binomial GLMM: *z* = −1.3, p = 0.192, n = 1004 observations of 28 individuals). Likewise, the probability of being sighted alone vs. with at least one conspecific (within ≤5 m) did not differ between proactive and reactive individuals (binomial GLMM: *z* = −0.592, p = 0.554, n = 1004 observations of 28 individuals).

## Discussion

We examined the relationship between coping style and space use within a well‐defined zoo habitat in a wild population of crows. Coping style was measured by docility and tonic immobility responses, and space use was quantified as the number of different sites visited within the zoo habitat.

We found that docility of crows was repeatable and significantly predicted their space use, with more reactive individuals visiting more sites, thus having a wider ranging space use within our study site. The reaction to the TI test was unstable over time in our study subjects and was thus disregarded for this study. We further controlled for a number of individual attributes that could potentially influence space use in crows. We took into account a possible effect of dominance, which has been reported to coincide with proactivity in other species (e.g. Verbeek et al. 1996; Bolhuis et al. 2005) but did not find a significant effect of the dominance‐related parameters of sex and age (Richner 1989) on space use. We also found no influence of body condition, hybridisation index or breeding status. The relationship between docility and space use in our study population appears robust, as it was found in both studies, independently of the definition of sites. These results were further confirmed by a common home range calculation using kernel density estimation.

A bias could arise from proactive crows generally visiting the zoo less than reactive ones, resulting in a smaller absolute space use within the zoo as a function of the number of visits. However, our data reject this hypothesis as we found no difference in the number of sightings between the behavioural types in study 1, and we found a contrary pattern in study 2, where proactive crows were sighted more often than reactive ones. Another potential bias is if proactive crows have home ranges that are predominantly outside of the zoo with only marginal overlap with the zoo area, as this would also result in a small space use within the zoo. Indeed, the location of the area within a habitat that individuals with different behavioural types occupy may differ, for example, depending on exposure to human disturbance (Martin & Réale 2008) or habitat quality (Duckworth 2006). However, if this was the case, we would expect proactive crows to be sighted on average closer to the periphery of the zoo than reactive ones, but there was no difference in that measure. Similarly, overlap with the zoo boundary did not differ between individuals with different coping styles.

Across many animal species, proactive individuals appear to fall into routines quickly, while reactive ones respond more flexibly to changes in the environment (reviewed in Koolhaas et al. 1999 and Wolf et al. 2008). This tendency to form habits could explain the space use patterns that we observed: if proactive crows rely on previous experience and stay within established routines, they might end up using the same few sites within the zoo repeatedly. Reactive birds, on the other hand, may react more readily to environmental stimuli, such as food availability and social cues (e.g. the presence of feeding birds), leading to a more flexible and extensive space use. Recent studies suggest that reactive individuals use social information regarding feeding sites more than proactive ones (Kurvers et al. 2010; Aplin et al. 2014). In our study, the number of conspecifics present in the immediate vicinity of a crow (≤5 m radius) did not differ between the behavioural types, suggesting that the larger space use of reactive crows was not the result of such a bias. However, social dynamics can be complex and deserve more examination than the scope of this study allowed.

Yet, our results appear to contrast with several previous studies across different species, where in fact proactive individuals (measured in exploration and activity) showed a wider ranging space use (North American red squirrels: Boon et al. 2008; Siberian Chipmunks: Boyer et al. 2010; starlings: Minderman et al. 2010; great tits: van Overveld et al. 2011; van Overveld & Matthysen 2010). One reason for this apparent discrepancy may lie in the different measures used to characterise behavioural type. Animals are thought to differ on multiple ‘personality’ dimensions (e.g. the ‘big five’ in humans; John & Srivastava 1999), and the number and types of dimensions are thought to vary between species in accordance with their evolutionary ecology (Réale et al. 2007). While previous studies used exploration and activity measures, we used the response to a stressor to characterise behavioural types. Thus, the relationship between behavioural type and space use may depend upon the particular behavioural measures used, and the apparent contrast between previous studies and the current results may stem from such methodological factors. Note, however, that in the red squirrel study population examined by Boon et al. (2008), individuals showing higher activity were also less docile during handling (i.e. more struggle) (Boon et al. 2007).

Alternatively, the contrast between our crow data and previous findings on great tits, starlings, North American red squirrels and Siberian chipmunks may indicate that the relationship between behavioural type and space use may be taxon specific and determined by social, ecological and evolutionary factors. If so, at present, we can only speculate which factors might be important in shaping this relationship. For example, both rodent species previously studied are territorial (Boon et al. 2008; Boyer et al. 2010), and individuals with wider space use often need to trespass neighbouring territories, possibly explaining why bolder and more aggressive individuals show greater space use in those species. In contrast, the crows we studied are socially quite tolerant (Miller et al. 2014) and most of our subjects were non‐breeders lacking defined territories. The study area included only a few nesting territories of breeding pairs, some of which were not even strongly defended. Interestingly, in great tits and starlings, the strength of the relationship between behavioural type and space use depended on age and habitat quality, respectively, indicating that this relationship is subject to context and environment. Differences in species‐specific socioecology may thus be an important characteristic to consider in future studies on personality‐dependent space use.

Variation in space use between individuals is likely to have multiple fitness implications. The spatial structure of a population is related to its social structure; thus, the location and size of the area utilised by individuals influences the frequency at which they encounter (particular) conspecifics (Brown & Orians 1970; Wolf et al. 2007). Further, both space use and social encounters are related to the acquisition and transmission of diseases and information. Behavioural types that come into contact with more locations and conspecifics may be more prone to pick up diseases, but could benefit from increased information gain (Barber & Dingemanse 2010; Boyer et al. 2010). Space use also determines access to resources such as food (Stephens & Krebs 1986) or shelter, and therefore may promote individual differences in foraging success and diet (Wilson 1998).

In summary, our study extends the currently limited literature on personality‐dependent local space use and suggests caution in generalising findings across species. We advocate considering personality‐dependent space use as a mediating factor between behavioural type and fitness. Our results further corroborate the increasing awareness that behavioural differences are an important factor to consider in studies and models concerning the spatial distribution of individuals within populations (Kurvers et al. 2010).

## Supporting information


**Figure S1:** Sketch of the zoo of Vienna (‘Tiergarten Schönbrunn’).
**Figure S2:** Division of the zoo into an evenly spaced hexagonal grid (grid sites) used to assess space use of crows.
**Figure S3:** Maps generated in QGIS, to identify the number of foraging sites (a) and grid sites (b) individual crows were sighted in, respectively.
**Table S1:** Frequency distribution of behavioural responses to coping style tests by all crows considered in the paper (n = 36).

**Video S1:** Crows' responses to the tonic immobility test.
