# Supplementary material for: Behavioural Type Affects Space Use in a Wild Population of Crows (Corvus corone)
Source: Ethology. 2016 Oct 6;122(11):881–91. doi: 10.1111/eth.12536 (PMC5082553; doi:10.1111/eth.12536)
Supplement: Supplementary file 1 — Figure S1: Sketch of the zoo of Vienna (‘Tiergarten Schönbrunn’). Figure S2: Division of the zoo into an evenly spaced hexagonal grid (grid sites) used to assess space use of crows. Figure S3: Maps generated in QGIS, to identify the number of foraging sites (a) and grid sites (b) individual crows were sighted in, respectively. Table S1: Frequency distribution of behavioural responses to coping style tests by all crows considered in the paper (n = 36). [file ETH-122-881-s001.pdf]

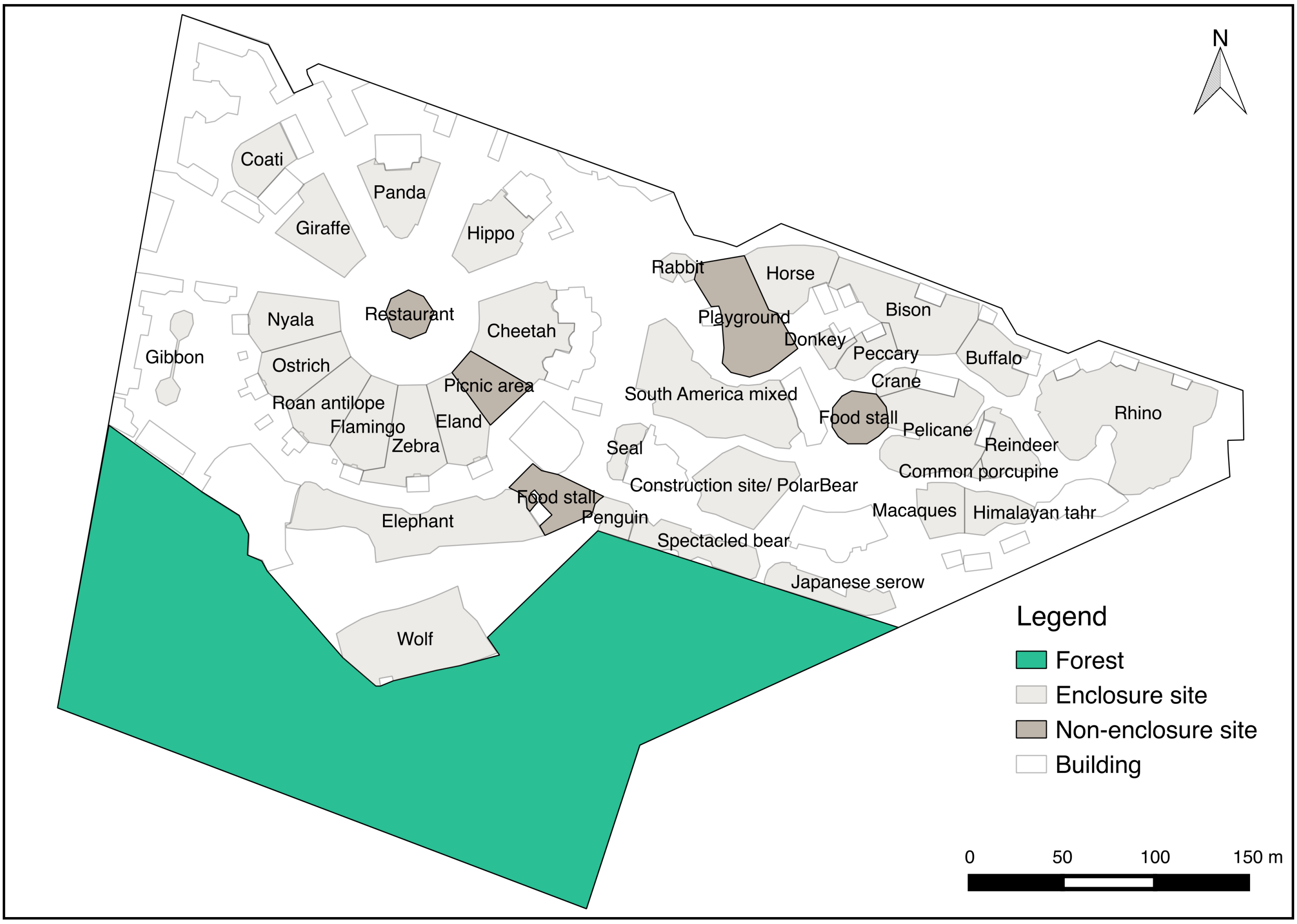

Figure S1: Sketch of the zoo of Vienna ("Tiergarten Schönbrunn"). The forested area was excluded from observations. The remaining area depicts the study site, with 38 foraging sites indicated. Enclosure sites (light grey) are labelled with the respective zoo-animal inhabitant, non-enclosure sites (dark grey) with an indication of their purpose.

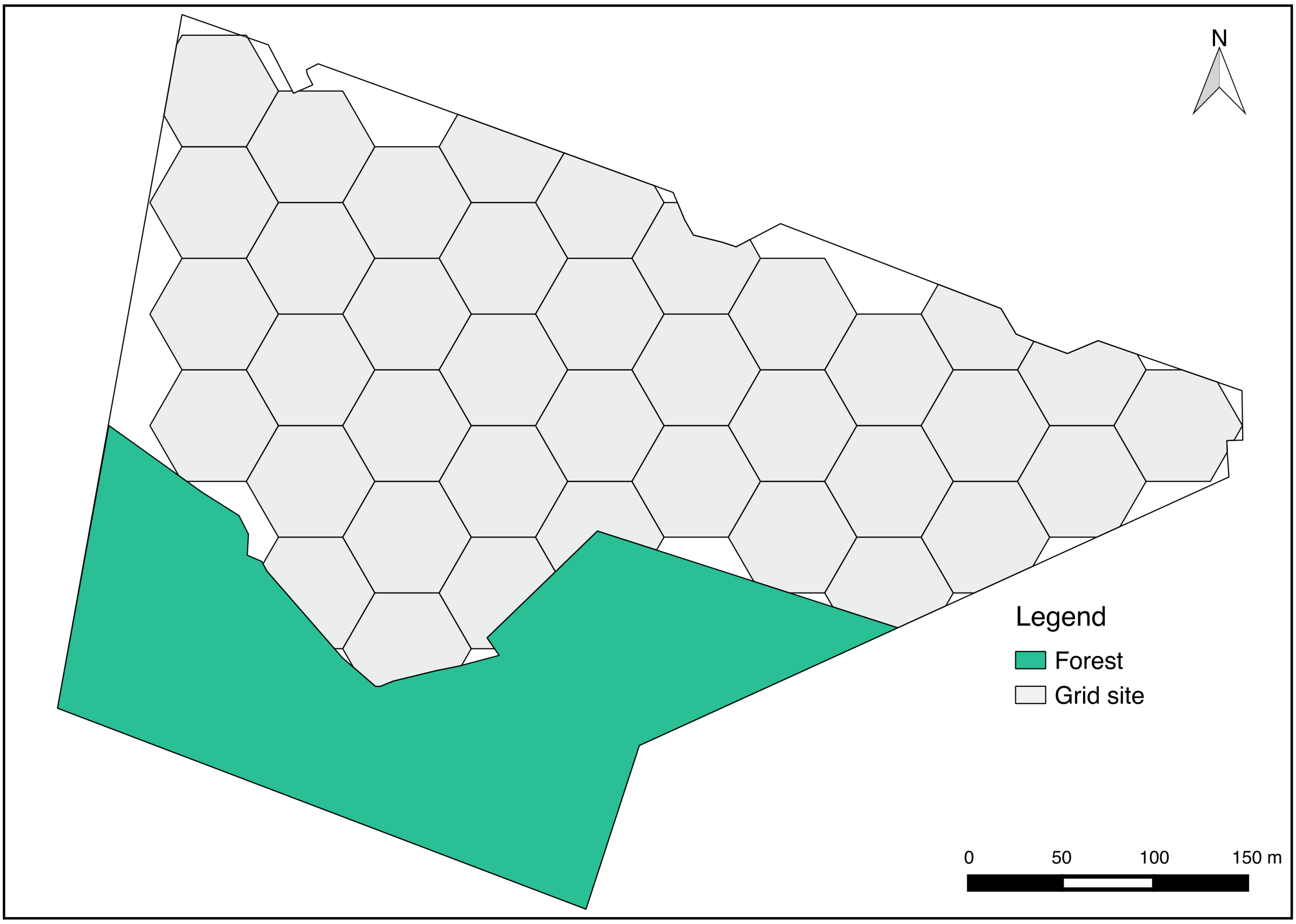

Figure S2: Division of the zoo into an evenly spaced hexagonal grid (grid sites) used to assess space use of crows.

(a)

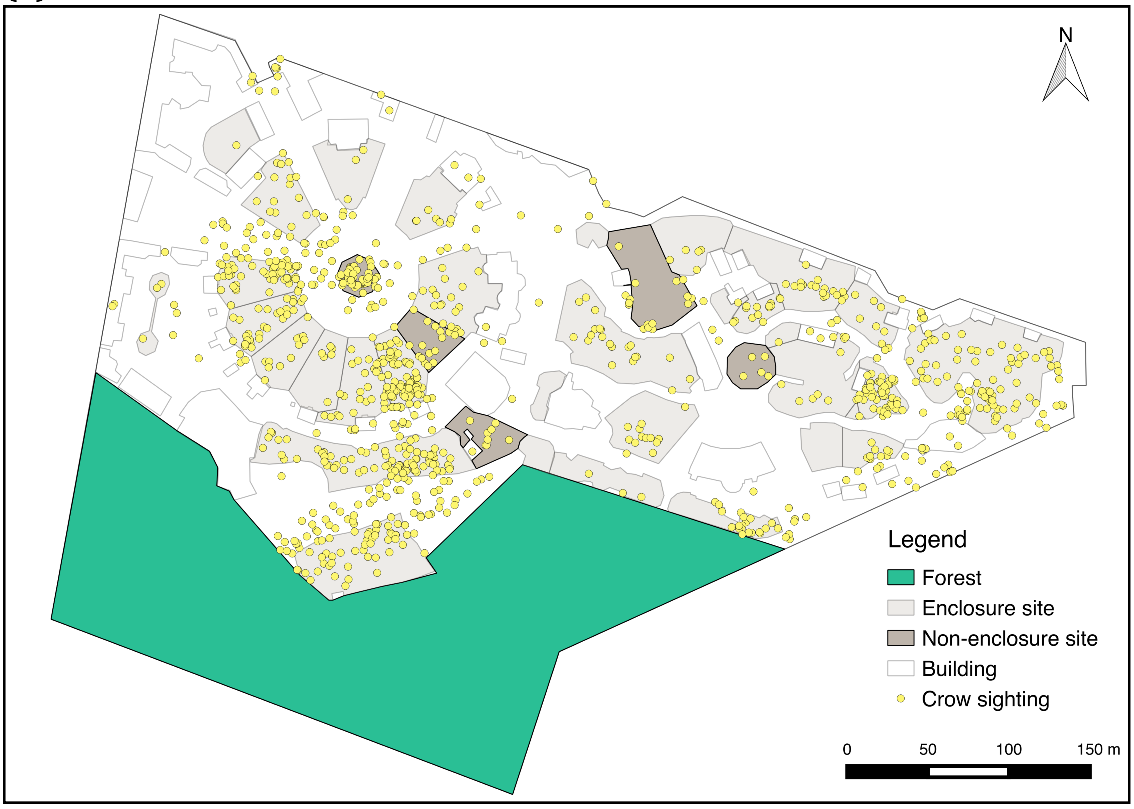

(b)

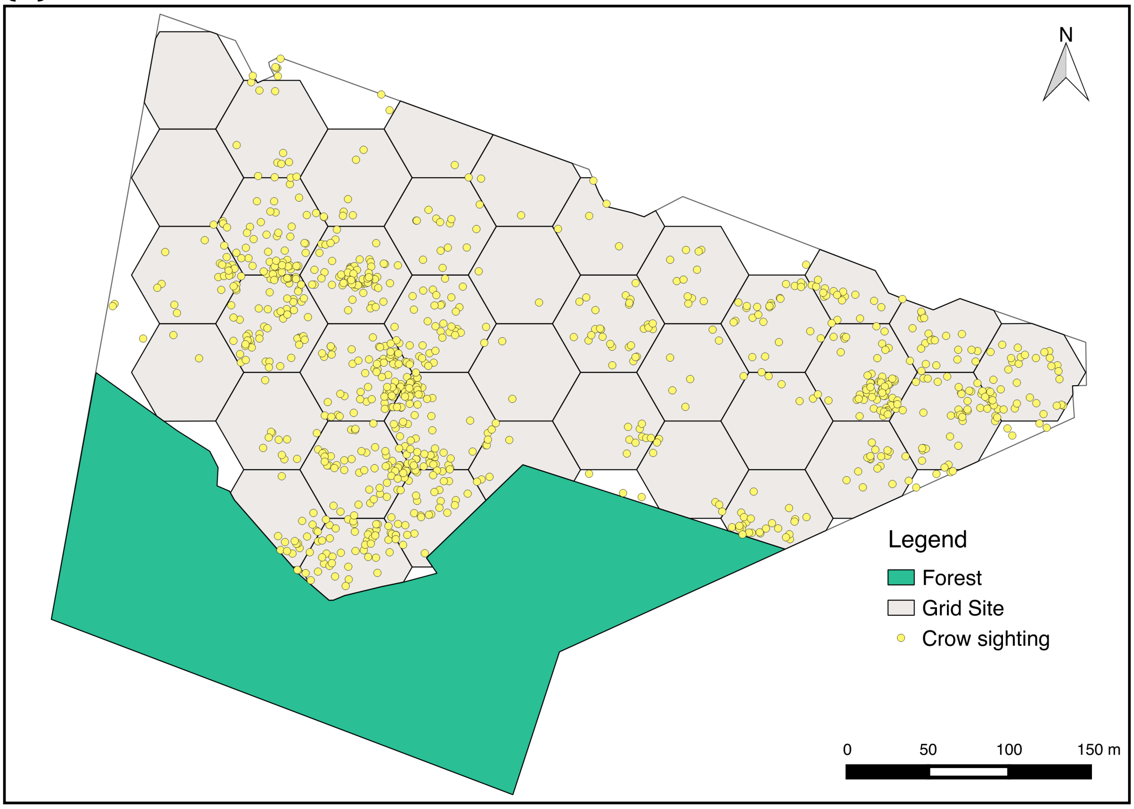

Figure S3: Maps generated in QGIS, to identify the number of foraging sites (a) and grid sites (b) individual crows were sighted in, respectively. Crow sightings in map (a) and (b) are identical, and depict all crow sightings from study 2 (n = 1004 sightings).

Table S1: Frequency distribution of behavioural responses to coping style tests by all crows considered in the paper (n = 36). Docility scores are given in 0 = no struggle and 1 = struggle during handling. Tonic immobility scores are given in 0 = TI induced and 1 = TI not induced.

		**Tonic immobility**		*Total*
		**0**	**1**	
**Docility**	**0**	11	17	*28*
	**1**	1	7	*8*
Total		*12*	*24*	*36*
